# Draft Genome Sequence of *Lactobacillus namurensis* Chizuka 01, Isolated from Nukadoko, a Pickling Bed of Fermented Rice Bran

**DOI:** 10.1128/genomeA.01263-13

**Published:** 2014-02-06

**Authors:** Keita Kato, Hidehiro Toh, Naoshige Sakamoto, Kazuki Mori, Kosuke Tashiro, Naruhiro Hibi, Kenji Sonomoto, Jiro Nakayama

**Affiliations:** a Department of Bioscience and Biotechnology, Faculty of Agriculture, Kyushu University, Fukuoka, Japan; b Medical Institute of Bioregulation, Kyushu University, Fukuoka, Japan; c Central Research Institute, Mizkan Group Corporation, Handa, Aichi, Japan; d Bio-Architecture Center, Kyushu University, Fukuoka, Japan

## Abstract

*Lactobacillus namurensis* Chizuka 01 was isolated from nukadoko, which is a fermented rice bran bed traditionally used in Japan for pickling vegetables. Here, we report the first draft of an annotated genome sequence of this organism. This paper is the first published report of the genomic sequence of *L. namurensis*.

## GENOME ANNOUNCEMENT

Nukadoko is initially prepared by natural fermentation of brine rice bran. Well-fermented nukadoko can be repeatedly used for pickling vegetables and can be refreshed by adding fresh rice bran approximately once per month. The repetitive cycles are sometimes continued for a number of years, resulting in well-aged nukadoko. Such well-aged nukadoko harbors a complex microbiota consisting of a number of *Lactobacillus* species, including two dominant species, *Lactobacillus namurensis* and *Lactobacillus acetotolerans* (1). *L. namurensis* was found to grow rapidly but stops growing a few days after the refreshment of nukadoko, whereas *L. acetotolerans* was found to grow slowly but continues growing for several weeks (2). The pH decreases as *L. namurensis* grows, suggesting that the organism produces a significant amount of lactate, which might contribute to the preservation of nukadoko for long periods. *L. namurensis* was originally isolated from a traditional Belgian sourdough and belongs to the *Lactobacillus buchneri* group, with *Lactobacillus zymae*, *Lactobacillus acidifarinae*, and *Lactobacillus spicheri* being the closest relatives (3). *L. namurensis* Chizuka 01 was isolated from well-aged nukadoko that has been recycled for >100 years.

The Chizuka 01 genome was sequenced with a Roche 454 GS (FLX Titanium) pyrosequencing system. Genomic libraries containing 3-kb inserts were constructed and sequenced, yielding 582,366 sequences that provided 77-fold coverage from both ends of the genomic clones. The sequence reads were assembled with Newbler assembler version 2.7 software (454 Life Sciences), which generated 97 contigs with a total length of 2,593,418 bp. The G+C content is 52%, which is slightly higher than those of other lactobacilli (4). The assembled contigs were submitted to the Microbial Genome Annotation Pipeline (http://www.migap.org/). The draft genome of Chizuka 01 contains 2,442 predicted protein-coding genes, 3 rRNA operons, and 57 tRNA genes. Using clusters of orthologous groups from the NCBI COGs database, functions were assigned to 2,143 protein-coding genes. The Chizuka 01 genome carries three operons involved in compatible solute import: *opuA* for glycine betaine, *opuC* for l-carnitine/choline, and *pro* for l-proline glycine betaine. The products of these genes are presumed to enhance tolerance to high salinity. The genes are found also in *Lactobacillus plantarum* and *Lactobacillus brevis*, organisms often isolated from brine-pickled vegetables (5). The Kyoto Encyclopedia of Genes and Genomes (KEGG) pathway analysis showed the presence of the pentose phosphate pathway in Chizuka 01 instead of the Embden-Meyerhof-Parnas pathway, suggesting a heterolactic fermentation property of Chizuka 01 that is consistent with the phenotype of this strain. In addition, two copies of *xylT* and 10 copies of *xynT* were detected, which may be involved in the import of xylose and xyloside, respectively, suggesting the ability of Chizuka 01 to ferment hemicellulose-derived sugars.

### Nucleotide sequence accession numbers.

The draft genome sequence for *L. namurensis* Chizuka 01 has been deposited in the DDBJ/GenBank/EMBL database under the accession no. BAOT01000001 to BAOT01000097.
